# University Hospital of Clinical and Ophthalmic Surgery, Berlin

**Published:** 1833-02

**Authors:** 


### UNIVERSITY HOSPITAL OF CLINICAL AND OPHTHALMIC SURGERY, BERLIN.

### *Cure of Aneurism of the Thyroid Gland, by Ligature of the two superior Thyroid Arteries.*

This operation, although proposed at an earlier period, was firmly established on principle, and successfully performed in 1814 by P. von Walther. It was first performed in Berlin, in 1819, at my hospital. When the gland, as in some cases, must be removed, owing to its size impeding respiration, the operation for its total extirpation is rendered the more difficult and dangerous; and therefore the indications for putting Walther's method into practice should be sought after with the greater zeal: this method is peculiarly fitted for the removing of the aneurismal state of the gland, as is proved by the following case.

An officer of the Prussian army suffered, according to his own statement, during the last six years, but probably since his childhood, from an enlargement of the thyroid gland, which latterly rapidly increased in size, so as, from its great weight, &c., to render him wholly unfit for service. The pulsations of the thyroid arteries, which were considerably enlarged, could not only be felt, but seen distinctly; the surface of this soft, elastic, and uneven tumor was covered by numerous varicose veins of considerable size, and pulsation to a greater or less degree could be perceived by the least pressure on any part of the tumor. It extended from the superior margin of the thyroid cartilage down to the sternum, and its extent from side to side was such as to leave the mastoidea muscles uncovered only at the superior extremities: it protruded forwards to the same extent as the chin. The voice was greatly impaired, and much inconvenience to the head arose from it; even slightly sudden motions were attended with a painful difficulty of breathing.

Under these circumstances, my patient was determined to undergo whatever might diminish his sufferings; and accordingly I performed the operation on the 19th of July, 1831. I commenced the incision at about the anterior edge of that part of the mastoid muscle which corresponded with the superior thyroid artery, immediately over the tumor, and continued it about two inches backwards, dividing the skin and platysma together. Having separated the edges of the wound, I succeeded, with a blunt instrument, in exposing the trunk of the artery: it appeared to be three or four times larger than natural, and pulsated violently. On the application of the ligature, the pulsations of the artery ceased entirely, and those of the tumor were considerably diminished. The operation on the opposite side was performed in precisely the same manner: indeed, the pulsation did not cease to the same degree; but, after some minutes, a remarkable and a manifest flaccidity of the entire gland took place.

Both wounds healed by first intention, excepting, of course, the track of the ligatures. The ligature of the right side came away in eight days; that of the left in ten days. In proportion as the adhesive inflammation (*obliterende entründung*) extended itself from the parts operated upon to the gland, in the same ratio did the pulsation of the latter become more and more indistinct, and the circumference of the tumor, which itself felt firmer, was greatly lessened in extent.

On the eleventh day after the operation, arterial hemorrhage occurred, though the wound was scarcely so large as would admit a pea: the hemorrhage was of that kind which we see sometimes occur after operation for aneurism. It was stopped by pressure of the finger, and afterwards by a graduated compress applied by means of adhesive plaster; however, notwithstanding, it occurred periodically until the fourteenth day, five times. By means of the blood effused in the cellular tissue in and about the wound, a perfect cure ensued, and the patient was perfectly recovered on the fifth week. The tumor then had lost two thirds of its circumference, and pulsation had entirely ceased. The patient asserted that he was altogether free from every former inconvenience, and that he could wear the ordinary regimental collar without its causing, in any motion whatsoever, the slightest inconvenience; he therefore returned to his regiment at Magdeburg. A short time since (in February 1832,) eight months after the operation, I heard from him, relative to his continued enjoyment of good health.

In the only other case in which I performed this operation in 1819, its result is directly opposed to this latter case, the tumor having completely resisted the operation of tying the artery, and, from the extreme danger its extreme size caused to the life of the patient, it was necessary to remove it entirely. During the operation, the whole anterior side of the neck, with trachea and the right carotid artery, was exposed: a portion of the gland was situated behind the larynx, and thirty-five arteries were obliged to be tied before the hemorrhage could be stopped. (See *Tractatus de Glandula Thyroidea*, &c., by A. Hedenus, Leipsic, 1822, p. 276.)

The cause of such different results should next be inquired into. In the first case, that of 1819, the aneurismal condition was manifestly present, but only, as I conceive, as accompanying the enlargement of the gland; the enlargement of the vessels progressed equally with the hypertrophy: whereas, in the latter case, the enlargement of the vessels evidently predominated, so that the enlargement of the affected organ was only conditional thereto. The thyroid arteries, in both cases, were enlarged, but those of the latter considerably more so, judging from their stronger pulsations, and also that the pulsations were distinctly to be seen. Without doubt the pointed-out and symptomatically-established differences account principally for the contrary results of the operations.

The difference in the manner of performing the operations might have certainly contributed somewhat to it.

In the first case I only tied the stronger pulsating artery of one side: in the latter, one on each side, which caused a greater diminution in the supply of blood, and an inflammation of greater intensity and extent, owing to the additional incision and ligature.

